# Healthcare utilisation patterns for respiratory and gastrointestinal syndromes and meningitis in Msunduzi municipality, Pietermaritzburg, KwaZulu-Natal Province, South Africa, 2013

**DOI:** 10.7196/SAMJ.2019.v109i5.13024

**Published:** 2019-04-29

**Authors:** J M McAnerney, C Cohen, A L Cohen, S Tempia, S Walaza, K K Wong, J Im, F Marks, H Dawood, U Panzner, K H Keddy, C von Mollendorf

**Affiliations:** 1 Centre for Respiratory Diseases and Meningitis, National Institute for Communicable Diseases of the National Health Laboratory Service, Johannesburg, South Africa; 2 School of Public Health, Faculty of Health Sciences, University of the Witwatersrand, Johannesburg, South Africa; 3 Influenza Division, Centers for Disease Control and Prevention, Atlanta, Ga, USA; 4 Strategic Information Group, Expanded Programme on Immunization, Department of Immunization, Vaccines and Biologicals, World Health Organization, Geneva, Switzerland; 5 Influenza Program, Centers for Disease Control and Prevention, Pretoria, South Africa; 6 Department of Epidemiology, International Vaccine Institute, Seoul, Republic of Korea; 7 Department of Medicine, Pietermaritzburg Metropolitan Hospital, South Africa; 8 CAPRISA (Centre for the AIDS Programme of Research in South Africa), University of KwaZulu-Natal, Durban, South Africa; 9 Centre for Enteric Diseases, National Institute for Communicable Diseases of the National Health Laboratory Services, Johannesburg, South Africa; 10 Department of Clinical Microbiology and Infectious Diseases, Faculty of Health Sciences, University of the Witwatersrand, Johannesburg, South Africa

## Abstract

**Background.:**

Public health facilities are used by the majority of South Africans, and healthcare utilisation surveys have been a useful tool to estimate the burden of disease in a given area.

**Objectives.:**

To describe care-seeking behaviour in a periurban site with a high prevalence of HIV infection, as well as barriers to seeking appropriate healthcare.

**Methods.:**

We conducted a cross-sectional household survey in 22 wards of the Msunduzi municipality in KwaZulu-Natal Province, South Africa, from October to December 2013 using a simple random sample of households selected from a 2011 census enumeration. A primary caregiver/adult decision-maker was interviewed regarding demographic data as well as health status and recent self-reported episodes of selected illnesses and healthcare utilisation.

**Results.:**

Of the 2 238 eligible premises visited, 1 936 households (87%) with a total of 9 733 members were enrolled in the study. Of these, 635 (7%) reported one or more episodes of infectious illness during the study period. Public health clinics were most frequently consulted for all illnesses (361/635, 57%). Private healthcare (general practitioner, private clinic, private hospital) was sought by 90/635 of individuals (14%), only 13/635 (2%) reported seeking care from traditional healers, religious leaders or volunteers, and 71/635 (11%) did not seek any medical care for acute illnesses. Individuals in the lowest income group were more likely to seek care at public health facilities than those in the highest income group (70% v. 32%).

**Conclusions.:**

Public health facility-based surveillance may be representative of disease patterns in this community, although surveillance at household level shows that high-income individuals may be excluded because they were more likely to use private healthcare, and the proportion of individuals who died at home would have been missed by facility-based surveillance. Data obtained in such surveys may be useful for public health planning.

In South Africa (SA), surveillance for respiratory diseases, diarrhoea and meningitis has been used to define the epidemiology, aetiology and seasonality of these syndromes.^[1–5]^ Healthcare utilisation surveys have been used to provide meaningful interpretation of surveillance data.^[6–8]^ These surveys provide estimates of the proportion of illness episodes not captured by health facility-based surveillance, which is most commonly used to estimate the burden of disease. While facility-based surveillance provides more accurate diagnoses, it may underestimate the burden of disease by omitting individuals who do not seek care at formal healthcare facilities. In addition, there are several barriers to care seeking that include shortages of staff, equipment or drugs as well as distance from healthcare facilities and high travel costs^[9–11]^ and may lead to a proportion of people not seeking healthcare through clinics or hospitals. Healthcare utilisation surveys can be used to estimate this proportion and explore reasons for not seeking care.

## Objectives

Edendale Hospital and the associated healthcare clinic in Pietermaritzburg, KwaZulu-Natal (KZN) Province, SA, have been sentinel surveillance sites for severe chronic respiratory illness, severe acute respiratory infection (SARI), influenza-like illness (ILI), diarrhoea and meningitis for several years.^[3,4,12–14]^ The prevalence of HIV in KZN in 2012 was 16.9%.^[15]^ We chose this location to conduct a healthcare utilisation survey to describe healthcare-seeking behaviour in this periurban surveillance site related to respiratory disease, gastrointestinal disease and meningitis in order to calculate the incidence of disease. In addition, we sought to identify barriers to seeking healthcare.

## Methods

### Study setting

Pietermaritzburg, the provincial capital of KZN, is situated ~80 km from the coastal city of Durban. The city falls within the Msunduzi local municipality, a primarily isiZulu-speaking area with a population of ~618 500 (2011 census).^[16]^ The area is served by a district hospital (Northdale Hospital), a referral hospital providing tertiary services (Grey’s Hospital) and Edendale Hospital (EDH), a large regional public hospital with 900 beds, making it the fourth-largest hospital in SA. EDH has served as a SARI surveillance site since 2009. It serves the surrounding neighbourhoods and townships of ~200 000 people. In addition, residents receive healthcare from a number of primary healthcare clinics referring to EDH, including the Edendale Gateway Clinic, where outpatient ILI surveillance is conducted.

### Study design and sampling

We conducted the survey in 22 of 38 wards of the Msunduzi local municipality, with a population of 361 582 in 63 008 households according to the 2011 census.^[16]^ A simple random sample of households throughout the catchment area of EDH was selected from a 2011 census enumeration, and all living household members of any age, as well as household members who had died within the previous 12 months, were enrolled in the study. A household was defined as all persons who live in the selected dwelling at least one night every week (excluding guests and temporary visitors), or a person or a group of related or unrelated persons living together in the same dwelling unit, who share the same housekeeping arrangements, and who provide themselves with food or other essentials for living. A household may be located in a housing unit or in a set of collective living quarters. Collective living arrangements where housekeeping arrangements and food are not shared were not considered as households.

The study was conducted from October to December 2013 after the end of the annual influenza season in SA.^[1]^ The catchment area was based on official referral patterns for the preceding year and data on the place of residence of patients treated at EDH. A sample of 1 614 households would enable us to estimate the proportion of individuals with an episode of pneumonia who utilised healthcare services with an absolute precision of 10%, assuming that 50% of people with pneumonia seek healthcare, a non-response rate of 15%, an average household size of 3.5 and a design effect of 2. The number of households was inflated to 2 400 to correct for non-residential or non-existing premises.^[16]^

Questionnaires (piloted, pre-tested and structured, with open- and closed-ended questions) were administered to respondents by one of 12 interview teams, each of which consisted of two lay community workers fluent in English and isiZulu.^[17]^ Interviewers underwent training prior to the start of the survey. All forms were translated into isiZulu, and back-translated to identify translation errors. Households were identified using geographical positioning system (GPS) and satellite maps. For each household, household composition data were collected from the primary caregiver/adult decision-maker, identified as the person most involved in the daily care of the household members. Health-seeking behaviour was ascertained separately for each individual. For young children this information was obtained from the parent or legal guardian. For individuals who had died or were absent at the time, it was collected from the head of the household. Teams visited households up to three times on separate days in order to enumerate members of the household and interview household members in person about demographic characteristics, medical conditions, selected illnesses and healthcare utilisation. Interviews were conducted in the preferred language of the household. Consent documents were provided in English or the preferred local language (isiZulu). Demographic data including household size, level of education, occupation and income were collected (the SA rand/US dollar exchange rate at the time of the study was ZAR1 to USD0.07), as well as health status, which included underlying conditions such as tuberculosis (TB) and HIV serostatus. Episodes of respiratory illnesses and meningitis and data on diarrhoea in children aged <5 years were also ascertained.

### Case definitions of illnesses surveyed

Study participants were asked whether they had any of the following clinical syndromes, which had been used in previous surveys.^[18]^

- **ILI.** Within the past 30 days, sudden-onset fever or worsening fever (measured temperature >38°C or subjective) with cough and/or sore throat
- **Acute pneumonia.** Within the past 1 year, diagnosis of pneumonia by a healthcare worker, or sudden onset of worsening fever (measured temperature >38°C or subjective) and cough and difficult breathing lasting 2 – 30 days
- **TB/chronic febrile respiratory illness** (a proxy for TB). Within the past year, fever and cough and either difficult breathing or weight loss lasting ≥30 days
- **Meningitis.** Within the past year, fever or headache, and one of the following: stiff neck, confusion, new weakness in arm or leg, or double vision
- **Diarrhoea.** For children aged <5 years, within the past 14 days, three or more loose or watery stools within a 24-hour period.

### Ethical considerations

The purpose of the study was explained in the preferred language of an adult primary caregiver. Written consent was provided by participating caregivers/respondents on behalf of all household members to collect household-level data. For those unable to write, a thumb print served in place of a signature. However, for individual-based data, verbal consent was obtained from all household members aged ≥18 years, and from the primary caregiver/respondent for individuals not present during the interview and for those aged <18 years.

Ethical approval for the study was obtained from the University of KwaZulu-Natal (ref. no. BE209/13). The study was determined to be within the scope of public health practice by the Centers for Disease Control and Prevention (non-research determination no. 2012 6165).

### Data management and statistical analysis

Data were entered on paper forms by the interviewers and sent to the National Institute for Communicable Diseases, where management was centralised. Clinical and demographic data from enrolled patients were recorded on a Microsoft Access 2010 (version 14.0) database (Microsoft, USA) with double data entry. All analyses were conducted using Stata version 14 (StataCorp, USA).

## Results

**Fig. 1.**
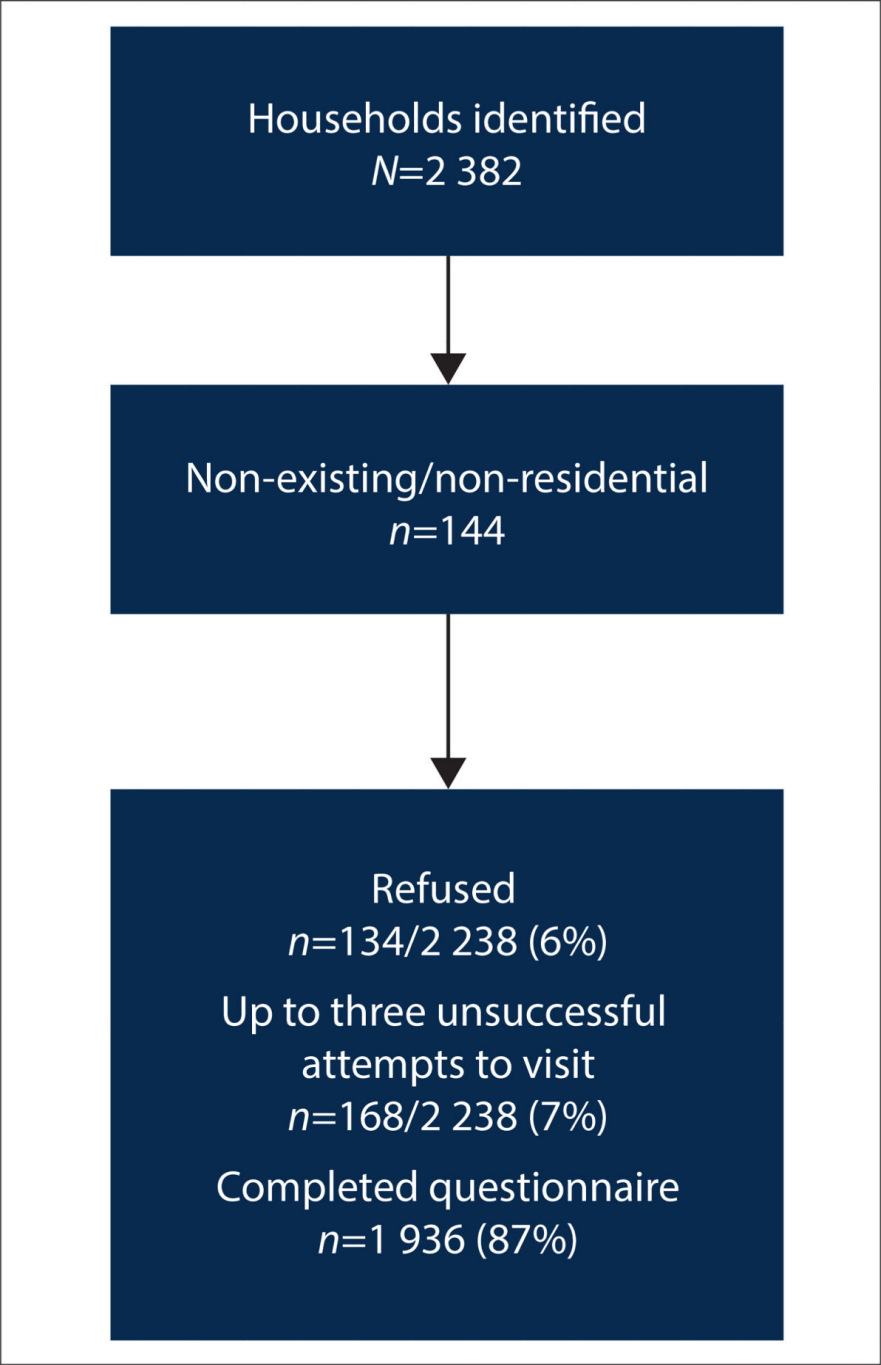
Households enrolled, Msunduzi municipality, Pietermaritzburg, South Africa, 2013.

Of the 2 400 randomly selected premises, 2 382 were visited (18 premises were not visited owing to time constraints). Of those visited, 144 (6%) were found to be either non-existent or non-residential and were excluded. Of the remaining 2 238 households visited, 134 (6%) households refused, and 168 (7%) were visited up to three times and either household members were not at home or no adult decision-maker was present. A total of 1 936 households (87%) were enrolled in the study (Fig. 1).

**Fig. 2.**
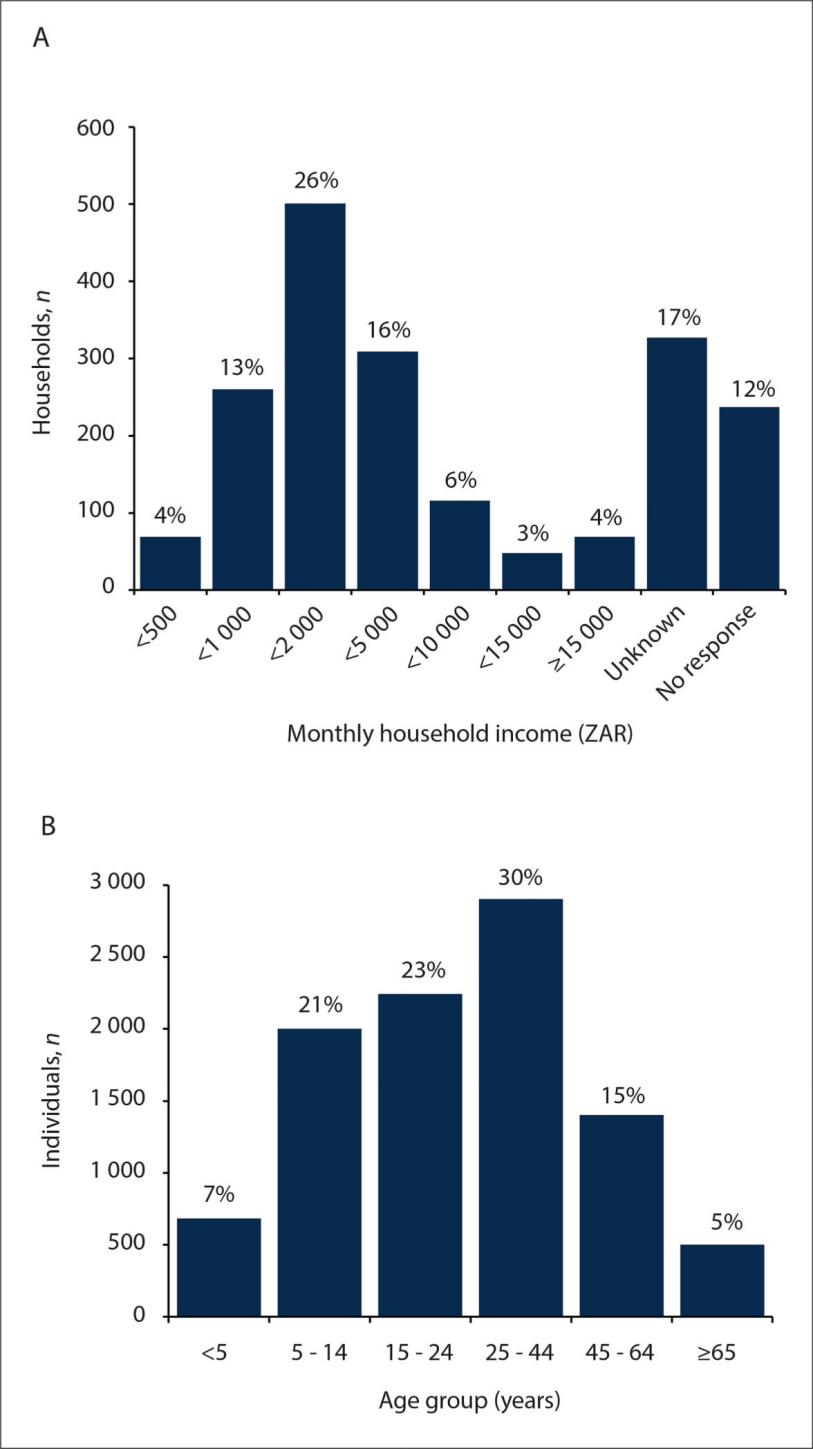
Characteristics of households and community members enrolled in a survey of healthcare utilisation, Msunduzi municipality, Pietermaritzburg, South Africa, 2013. A = monthly household income; B = age groups. The SA rand/US dollar exchange rate at the time of the study was ZAR1 to USD0.07.

Data were collected for 9 733 household members, 45% (4 373/9 733) of whom were male. Between 1 and 20 individuals lived in a household, with a median household size of 5 individuals (interquartile range 3 – 7) and a mean of 5.02. Ages ranged between 1 month and 105 years, with 30% aged 25 – 44 years. In the majority (1 434/1 936, 74%) of households the head of the household was unemployed and was not the main source of household income. For the 1 372/1 936 (71%) who responded, the median monthly household income was between ZAR1 000 and ZAR1 999 (USD70 – 135) with 23% (309/1 372) having an income between ZAR2 000 and ZAR4 999 (USD135 – 335) (Fig. 2). The prevalence of self-reported HIV infection was 7% in the 8 331/9 733 (86%) individuals who responded to this question (Table 1). The most frequently reported other underlying condition was diabetes (380/9 678, 4%), followed by asthma (314/9 638, 3%). The proportion of individuals with reported diabetes increased with age, as did the proportions reporting heart disease and asthma. The proportion of individuals with reported TB during the past year was 0.7% (66/9 733) and was highest in the 45 – 64-year age group (1.2%, 17/1 403), whereas the 25 – 44-year age group had the highest proportion (362/2 409, 15%) of individuals with self-reported HIV infection (Table 1).

Of the 2 927 women of childbearing age (15 – 49 years), 81 (3%) were pregnant, with the highest proportion (36/918, 4%) in the 25 – 34-year age group.

One or more episodes of infectious illness were reported in 635/9 733 (7%) household members in 492/1 936 (25%) households during the period of interest (Table 2). Pneumonia during the previous year was reported in 50/9 733 (0.5%) individuals, with the highest proportion in the 45 – 64-year age group (16/1 403, 1%), followed by children aged <5 years 7/683, 1%). Almost 5% (480/9 733) of individuals interviewed reported one or more episodes of ILI in household members during the preceding 30 days, with the highest proportion among children aged <5 years (55/683, 8%). TB/chronic febrile respiratory illness in the past year were reported in a total of 66/9 733 (0.7%) individuals, with the highest proportion (17/1 403, 1%) in adults aged 45 – 64 years. Meningitis was reported in 39/9 733 (0.4%) individuals, with the highest proportion in the age group ≥65 years (8/499, 2%). In addition, diarrhoea during the previous 2 weeks was reported in 21/682 (3%) of children aged <5 years.

The type of health facility first approached by the majority of individuals was a public clinic (333/557, 60%), followed by pharmacies (110/557, 20%) and general practitioners (63/557, 11%) (Table 3).

The type of health facility most frequently consulted for all illnesses was a public clinic (361/635, 57%) (Table 4). Individuals with TB/chronic febrile respiratory illness most commonly used public clinics (53/66, 80%). Of those attending a public clinic, 40/361 (11%) attended Edendale Clinic. Eighteen percent (114/635) of individuals attended or were referred to a public hospital, of whom 92/114 (81%) attended EDH. The highest proportion of those attending either a public or private hospital was among individuals with TB/chronic febrile respiratory illness (38/66, 58%), followed by pneumonia (27/50, 54%). Private healthcare (general practitioner, private clinic or hospital) was sought by 90/635 (14%) of individuals. Of those who sought care at a pharmacy, 107/132 (81%) did not seek care elsewhere; the majority of these (105/107, 98%) reported ILI. Very few individuals (13/635, 2%) reported seeking care from traditional healers, religious leaders or community health workers, and all subsequently sought care at a clinic or hospital. Among individuals reporting any illness, 71/635 (11%) did not seek medical care; 61/71 (86%) of these reported ILI.

More than half of the individuals with ILI who did not seek medical care (36/61, 59%) reported self-medicating. The most common reason given for not seeking care at all was that they did not feel that they or their child were sick enough. Other reasons included that the sick person was improving or that there was no one available to take him or her to a healthcare provider.

A higher proportion of individuals in the lowest income group of <ZAR1 000 (<USD70) per month (86/122, 70%) than individuals in the highest income group (23/71, 32%) reported using public healthcare; furthermore, the highest income group had the largest proportion of individuals using private healthcare (24/71, 34%). The largest proportion of individuals not seeking care (9/44, 20%) was in the ≥65-year age group, and the majority (6/9) of these reported ILI. HIV-infected individuals, who mainly reported pneumonia and/or chronic febrile respiratory illness (61/85, 72%), were more likely to report attending public health facilities (62/85, 73%) than HIV-uninfected individuals and those with unknown HIV status (269/468 (58%) and 47/82 (57%), respectively) (Table 5).

During the year preceding the survey, 92 household members were reported to have died. Their ages ranged from 3 months to 94 years (median 42 years), and 57 (62%) had died in hospital, 32 (35%) at home, 2 (2%) at the scene of an accident, and 1 (1%) on the way to hospital. None of the <15-year-olds who died (*n*=6) were known to be HIV-infected, but 14/31 (45%) of the 25 – 44-year-olds were reported to be HIV-infected (Table 6). Although 70/92 (76%) of households reported that they had a death certificate for the deceased, only 34/70 (49%) certificates were shown to the interviewers. Four of the individuals who died had sought care for chronic febrile respiratory illness during the study period, and 1 for an ILI. Death certificates were available for 3 of these patients, and recorded causes of death were pneumonia (HIV-positive), TB (HIV status unknown) and stroke (HIV-positive).

## Discussion

In this study we characterised patterns of health-seeking behaviour in a periurban community with a high prevalence of HIV. We found that public health facilities were most commonly consulted for all infectious syndromes studied, with most people seeking care at public clinics, followed by public hospitals. This may demonstrate trust in and acceptability of the public health system, but in contrast it may reflect that the public health system is the only affordable and accessible option for this population. Although 12% of individuals consulted a general practitioner, only a small proportion (3%) of individuals used private clinics or hospitals. In a similar survey carried out in Gauteng and North West provinces in SA in 2012, public healthcare facilities were also most commonly consulted, with a similar proportion of respondents consulting general practitioners; however, compared with our study, larger proportions of subjects consulted private clinics and hospitals (11% and 16%, respectively).^[18]^ Consultations with traditional healers and religious leaders for infectious syndromes were rare (2%) in our study compared with some previous surveys,^[6]^ but 21% of individuals consulted pharmacies, which dispense both allopathic and complementary medicines. Pharmacies may well be an appropriate option for ILI. Importantly, more affluent people were less likely to use public health facilities, which suggests that surveillance based at public hospitals may not be fully representative of higher income groups.

Only 13% of households selected for the study did not participate because the head of the household refused to participate (6%) or there was no adult caregiver to be interviewed during up to three visits, which was similar to non-participation rates in North West, but lower than those in Gauteng.^[18]^ Where income was known, in most households (830/1 372, 60%) it was <ZAR2 000 (USD135) per month, and in the majority of households the head of the household was unemployed.

The finding of a mean household size of 5 when we had estimated a mean of 3.5 led to a larger sample size, giving more power to the study.

Analysis of self-reported underlying conditions showed that the proportions of individuals with diabetes, asthma, heart disease and TB increased with age, as documented elsewhere.^[19]^ The proportion of self-reported HIV was highest in the 25 – 44-year age group at 15%, compared with 28% confirmed HIV positivity using dried blood spots in the 15 – 49-year-age group in a population survey in KZN in 2012.^[15]^

One or more infectious syndromes were reported in 7% of household members. Acute ILI was most commonly reported in young children, while pneumonia and chronic febrile respiratory illness, although relatively rare, occurred mainly in the 45 – 64-year age group. The majority of individuals with serious illnesses sought help from hospitals rather than primary care clinics, indicating a measure of health literacy and appropriate healthcare-seeking behaviour. Reported healthcare-seeking behaviour varied by income level, age group and reported HIV status. A larger proportion of individuals in the highest income group sought private healthcare compared with the two lower income groups and those with unknown income. The proportion of self-reported HIV-positive individuals seeking care at public health facilities was higher than that of uninfected individuals, which may point to trust in the public healthcare system, although there is continuing stigma around HIV infection.

Only 8½ 927 (3%) of the women of childbearing age (15 – 49 years) were pregnant, a lower rate than that in the 2013 mid-year population estimates, which estimated the pregnancy rate for women aged 15 – 49 years at 9%.^[20]^

Although 57/92 (62%) of the individuals who had died during the year preceding the survey died in hospital, 32/92 (35%) died at home. In SA it was found that 44% of all-cause deaths occurred at home, though the proportion was lower in the age group <5 years. Importantly, these deaths would be missed by hospital-based surveillance.^[21]^

The findings of our study reflect one point in time. We were unable to find previous data on trends in heath-seeking behaviour for the syndromes studied, and it would be of interest to examine the trends in the future.

### Study limitations

Our study has several limitations. All illnesses were self-reported, either by the individual or the head of household, so it is possible that the case definitions were inaccurate. The definition for meningitis included symptoms of chronic meningitis, which can be diagnostically nonspecific, so it is possible that some illnesses classified as meningitis were not meningitis. Also, the case definitions for both meningitis and pneumonia required a recall period of up to 1 year by the patient or the adult caregiver, and it is probable that more recent or very severe episodes were more likely to be captured. Adult caregivers may also have been unaware of mild illness or HIV status. The prevalence of self-reported HIV in this study was ~40% of estimates obtained in the national HIV survey of 2012, suggesting reporting bias or that people may not have accessed HIV testing;^[15]^ there is also still substantial stigma associated with HIV infection. In addition, the reported pregancy rate was a third of the national estimated pregancy rate, suggesting under-reporting for all illnesses. Very few individuals reported consulting traditional healers, which differed from a study in rural Kenya.^[6]^ Although the interview team consisted of lay community workers, the low rate of traditional healer consultation may have represented reporting bias. We are also unable to generalise on health-seeking behaviour beyond the syndromes surveyed.

## Conclusions

This study shows that the majority of patients in the area surveyed sought care for the syndromes surveyed at public clinics and public hospitals in the area, suggesting that public health facility-based surveillance may capture a large proportion of episodes of illness in the community, although high-income individuals may be selectively excluded, as this group was more likely to utilise private healthcare. However, a large proportion of individuals who died at home would have been missed by facility-based surveillance. These data in conjunction with surveillance data can be used to generate estimates of disease burden that may be useful for public health planning.

## Figures and Tables

**Table 1.** Self-reported underlying conditions by age group in community members enrolled in a survey of healthcare utilisation, Msunduzi municipality, Pietermaritzburg, South Africa, 2013

Age group (years)	Diabetes, *n/N* (%; 95% CI)	Asthma, *n/N* (%; 95% CI)	Heart disease, *n*/*N* (%; 95% CI)	Tuberculosis, *n*/*N* (%; 95% CI)	HIV, *n/N* (%; 95% CI)

<5	0/682	19/681 (2.8; 1.8 – 4.3)	2/681 (0.3; 0.07 – 1.2)	2/681 (0.3; 0.07 – 1.2)	1/610 (0.2; 0.02 – 1.1)
5 – 14	2/1 991 (0.1; 0.03 – 0.4)	59/1 984 (3.0; 2.3 – 3.8)	10/1 993 (0.5; 0.3 – 0.9)	6/1 993 (0.3; 0.1 – 0.7)	39/1 787 (2.2; 1.5 – 3.1)
15 – 24	4/2 235 (0.20; 0.06 – 0.4)	55/2 218 (2.5; 1.9 – 3.2)	17/2 235 (0.7; 0.4 – 1.1)	11/2 237 (0.5; 0.3 – 0.9)	68/1 905 (3.6; 2.8 – 4.5)
25 – 44	50/2 883 (1.7; 1.3 – 2.3)	80/2 872 (2.8; 2.2 – 3.5)	36/2 877 (1.3; 0.9 – 1.7)	50/2 876 (1.7; 1.3 – 2.3)	362/2 409 (15.0; 13.4 – 16.8)
45 – 64	215/1 394 (15.4; 13.6 – 17.4)	73/1 390 (5.3; 4.1 – 6.6)	96/1 399 (6.9; 5.6 – 8.3)	41/1 398 (3.0; 2.1 – 4.0)	101/1 189 (8.5; 7.0 – 10.3)
≥65	109/493 (22.1; 18.6 – 26.1)	28/493 (5.7; 3.9 – 8.1)	46/494 (9.3; 7.0 – 12.2)	11/494 (2.2; 1.2 – 4.2)	7/431 (1.6; 0.7 – 3.4)
Total	380/9 678 (3.9; 3.5 – 4.3)	314/9 638 (3.3; 2.8 – 3.7)	206/9 679 (2.1; 1.8 – 2.5)	121/9 679 (1.3; 1.0 – 1.5)	578/8 331 (7.0; 6.3 – 7.7)

CI = confidence interval.

**Table 2.** Illness reported by age group in community members enrolled in a survey of healthcare utilisation, Msunduzi municipality, Pietermaritzburg, South Africa, 2013*

Age group (years)	Total responding, *N*	Pneumonia, *n* (%; 95% CI)	ILI, *n* (%; 95% CI)	Tuberculosis/chronic respiratory illness, *n* (%; 95% CI)	Meningitis, *n* (%; 95% CI)

<5	683	7 (1.0; 0.5 – 2.0)	55 (8.1; 6.2 – 10.6)	3 (0.4; 0.1 – 1.3)	0
5 – 14	2 000	7 (0.4; 0.1 – 0.7)	100 (5.0; 4.0 – 6.1)	3 (0.2; 0.04 – 0.4)	1 (0.1; 0.07 – 0.4)
15 – 24	2 244	4 (0.2; 0.07 – 0.6)	86 (3.8; 3.1 – 4.7)	10 (0.4; 0.2 – 0.8)	4 (0.2; 0.07 – 0.5)
25 – 44	2 904	12 (0.4; 0.2 – 0.7)	125 (4.3; 3.5 – 5.1)	30 (1.0; 0.7 – 1.5)	15 (0.5; 0.3 – 0.8)
45 – 64	1 403	16 (1.1; 0.7 – 1.8)	85 (6.1; 4.9 – 7.5)	17 (1.2) (0.7 – 2.0)	11 (0.8; 0.4 – 1.4)
≥65	499	4 (0.8; 0.3 – 2.1)	29 (5.8; 4.0 – 8.4)	3 (0.6; 0.2 – 1.8)	8 (1.6; 0.8 – 3.2)
Total	9 733	50 (0.5; 0.4 – 0.7)	480 (4.9; 4.5 – 5.4)	66 (0.7; 0.5 – 0.9)	39 (0.4; 0.3 – 0.5)

CI = confidence interval; ILI = influenza-like illness.

* Time period for pneumonia, tuberculosis/chronic respiratory illness and meningitis – within the past year; ILI – within the past 30 days.

**Table 3.** Order in which healthcare was sought by community members enrolled in a survey of healthcare utilisation, Msunduzi municipality, Pietermaritzburg, South Africa, 2013

Facility	First visit (*N*=557), *n* (%)	Second visit (*N*=130), *n* (%)	Third visit or more (*N*=33), *n* (%)

Public clinic	333 (59.8)	18 (13.8)	2 (6.1)
Pharmacy	110 (19.8)	10 (7.7)	7 (21.2)
General practitioner	63 (11.3)	21 (16.2)	6 (18.2)
Public hospital	26 (4.7)	73 (56.2)	7 (21.2)
Private clinic	8 (1.4)	2 (1.5)	0
Private hospital	4 (0.7)	3 (2.3)	0
Other*	13 (2.3)	3 (2.3)	11 (33.3)

* Traditional healer, religious leader, community health worker.

**Table 4.** All healthcare facilities and providers* consulted by community members reporting illness enrolled in a survey of healthcare utilisation, Msunduzi municipality, Pietermaritzburg, South Africa, 2013

Facilities and providers	All illness (*N*=635), *n* (%; 95% CI)	Pneumonia (*N*=50), *n* (%; 95% CI)	ILI (*N*=480), *n* (%; 95% CI)	Tuberculosis/chronic respiratory illness (*N*=66), *n* (%; 95% CI)	Meningitis (*N*=39), *n* (%; 95% CI)

Public clinic	361 (56.9; 51.4 – 61.0)	29 (58.0; 43.6 – 71.2)	256 (53.3; 48.5 – 58.3)	53 (80.3; 68.3 – 88.5)	23 (59.0; 47.3 – 77.9)
Edendale Clinic	40 (6.3)	3 (6.0)	28 (5.8)	5 (7.6)	4 (10.3)
Pharmacy	132 (20.8; 17.6 – 24.7)	2 (4.0; 1.0 – 15.3)	126 (26.3; 21.9 – 30.8)	1 (1.5; 0.2 – 10.5)	3 (7.7; 3.7 – 25.2)
Public hospital	114 (18.0; 14.6 – 20.9)	25 (50.0; 36.0 – 63.9)	37 (7.7; 5.6 – 11.0)	38 (57.6; 44.8 – 69.3)	14 (35.9; 22.0 – 52.6)
Edendale Hospital	92 (14.5)	18 (36.0)	29 (6.0)	33 (50.0)	12 (30.8)
General practitioner	74 (11.7; 9.4 – 15.1)	14 (28.0; 17.0 – 42.4)	50 (10.4; 8.1 – 14.5)	6 (9.1; 4.0 – 19.3)	4 (10.3; 5.2 – 28.2)
Did not seek care	71 (11.2; 8.9 – 14.2)	2 (4.0; 1.0 – 15.2)	61 (12.7; 9.8 – 16.2)	1 (1.5; 0.2 – 10.5)	7 (17.5; 8.5 – 33.9)
Private clinic	10 (1.6; 0.7 – 2.8)	1 (2.0; 0.2 – 3.1)	7 (1.5; 0.7 – 3.0)	1 (1.5; 0.2 – 10.5)	1 (2.6)
Private hospital	6 (0.9; 0.3 – 1.9)	2 (4.0; 0.9 – 1.5)	3 (0.6; 0.2 – 1.9)	0	1 (2.6)
Other^†^	13 (2.0; 0.9 – 2.4)	-	-	-	-

CI = confidence interval; ILI = influenza-like illness.

* More than one facility or provider could be consulted. Data for Edendale Clinic and Edendale Hospital are subsets of Public clinic and Public hospital – confidence intervals were not calculated for these estimates.

† Traditional healer, religious leader, community health worker.

**Table 5.** All healthcare facilities and providers consulted by community members reporting illness enrolled in a survey of healthcare utilisation, Msunduzi municipality, Pietermaritzburg, South Africa, 2013, by income, age group and HIV status

	Income group (ZAR*), *n* (%)				HIV, *n* (%)			Age group (years), *n* (%)		
Facilities	<1 000 (*N*=122)	1 000 – 4 999 (*N*=284)	≥5 000 (*N*=71)	Unknown (*N*=158)	Infected (*N*= 85)	Uninfected (*N*=468)	Unknown (*N*=82)	<5 (*N*=66)	5 – 64 (*N*=525)	≥65 (*N*=44)

Public health facilities	86 (70.5)	167 (58.8)	23 (32.4)	102 (64.7)	62 (72.9)	269 (57.5)	47 (57.3)	42 (63.6)	312 (59.4)	24 (54.6)
Private healthcare	8 (6.6)	26 (9.2)	24 (33.8)	14 (8.9)	3 (3.5)	60 (12.8)	9 (11.0)	9 (13.6)	55 (10.5)	8 (18.2)
Pharmacy	18 (14.8)	55 (19.4)	14 (19.7)	27 (17.1)	13 (15.3)	84 (18.0)	17 (20.7)	10 (15.2)	101 (19.2)	3 (6.8)
No care	10 (8.2)	36 (12.7)	10 (14.1)	15 (9.5)	7 (8.2)	55 (11.8)	9 (11.0)	5 (7.6)	57 (10.9)	9 (20.5)

* South African rand/US dollar exchange rate at the time of the study ZAR1 to USD0.07.

**Table 6.** Deaths of community members enrolled in a survey of healthcare utilisation, Msunduzi municipality, Pietermaritzburg, South Africa, 2013, by age group and reported underlying conditions

Age group (years)	Total, *n/N* (%; 95% CI)	Diabetes, *n* (%*; 95% CI)	Asthma, *n* (%*; 95% CI)	Heart disease, *n* (%*; 95% CI)	Tuberculosis, *n* (%*; 95% CI)	HIV, *n* (%*; 95% CI)

<5	5/682 (0.7; 0.02 – 1.3)	0	0	0	0	0
5 – 14	0/1 991	0	0	0	0	0
15 – 24	7/2 235 (0.3; 0.1 – 0.7)	0	1 (14.3; 0.36 – 57.8)	0	1 (14.3; 0.36 – 57.8)	2 (28.6; 3.7 – 70.9)
25 – 44	36/2 883 (1.2; 0.9 – 1.7)	1 (2.8) (0.7 – 14.5)	2 (5.6; 0.7 – 18.6)	0	8 (22.2; 10.1 – 39.2)	14 (38.9; 23.1 – 56.5)
45 – 64	24/1 394 (1.7; 1.0 – 2.3)	5 (20.8) (7.1 – 42.1)	4 (16.7; 4.7 –37.3)	2 (8.3; 1.0 – 26.9)	9 (37.5; 16.5 – 53.9)	4 (16.7; 4.7 – 37.3)
≥65	20/493 (4.1; 2.8 – 6.4)	6 (30.0) (11.9 – 54.3)	1 (5.0; 0.1 – 24.9)	2 (10.0; 0.1 – 31.6)	1 (5.0; 0.1 – 24.9)	1 (5.0; 1 – 24.9)
Total	92/9 733 (0.9; 0.7 – 1.1)	12 (13.8) (6.9 – 21.7)	8 (9.2; 3.8 – 16.4)	4 (4.6; 1.2 – 10.8)	19 (21.8; 12.9 – 30.4)	21 (24.1; 14.7 – 32.7)

CI = confidence interval.

* Percentage of total deaths in age group.
